# Identification of hub genes and pathways of ferroptosis in *Fusarium* keratitis by bioinformatics methods

**DOI:** 10.3389/fcimb.2023.1103471

**Published:** 2023-01-31

**Authors:** Xingbo Teng, Xuewei Xiong, Xiaoyuan Sha, Yahui Lei, Yuyao Diao, Jiayan Liu, Yuan Tian, Lian Liu, Jingxiang Zhong

**Affiliations:** ^1^ Department of Ophthalmology, The First Affiliated Hospital of Jinan University, Jinan University, Guangzhou, China; ^2^ The Sixth Affiliated Hospital of Jinan University, Jinan University, Dongguan, China

**Keywords:** fungal keratitis, *Fusarium* keratitis, ferroptosis, bioinformatics, immune

## Abstract

**Background:**

Fungal keratitis is a common blinding eye disease, and Fusarium is one of the main species that cause fungal keratitis. As is well known, oxidative stress plays an important role in Fusarium keratitis and it is also a significant initiating factor of ferroptosis. But the relationship between Fusarium keratitis and ferroptosis is currently unclear. This study aimed to speculate and validate potential ferroptosis-related genes in Fusarium keratitis using bioinformatics analysis, which provided ideas for further research on its specific mechanism and new targets for its treatment.

**Methods:**

The microarray expression profiling dataset (GSE58291) came from Gene Expression Omnibus (GEO). The differentially expressed genes (DEGs) were obtained by the limma package of the R software. The DEGs were performed by Gene Ontology (GO) and Kyoto Encyclopedia of Genes and Genomes (KEGG) enrichment analysis. Then, the DEGs were intersected with the genes in the ferroptosis database. The top 5 hub genes were obtained by the protein-protein interaction (PPI) network analysis and the cytoHubba plug-in of Cytoscape software. The hub genes were subjected to GSEA analysis. Then we analyzed the immune infiltration of the samples by CIBERSORT and ssGSEA algorithm. Finally, we validated the mRNA of hub genes by qPCR.

**Results:**

A total of 1,368 DEGs were identified and 26 ferroptosis-related DEGs were obtained. At the same time, ferroptosis-related pathways were enriched by GO and KEGG using DEGs. HMOX1, CYBB, GPX2, ALOX5 and SRC were obtained by the PPI network analysis and the cytoHubba plug-in of Cytoscape software. The iron metabolism and immune response related pathways were enriched using GSEA. They included hematopoietic cell lineage, lysosome and FC gamma R mediated phagocytosis. T cells follicular helper, monocytes, macrophages and mast cells might play an important role in Fusarium keratitis using analysis of immune infiltration. Finally, qPCR confirmed that the expression of HMOX1, CYBB, ALOX5 mRNA in the DON group was significantly elevated, while the expression of GPX2 were significantly decreased.

**Conclusions:**

Ferroptosis may play an important role in Fusarium keratitis. HMOX1, CYBB, ALOX5 and GPX2 may be key ferroptosis-related genes in the pathogenesis of Fusarium keratitis.

## Introduction

Fungal keratitis is one of the major eye diseases in the world, especially in tropical and subtropical regions (Srinivasan, 2004). It is also one of the strongest blinding diseases (Thomas et al., 2005; Burton et al., 2011). Wearing contact lenses and plant trauma were major factors (Iyer et al., 2006; Ahearn et al., 2008; Gower et al., 2010). In fungal keratitis, *Fusarium* and *Aspergillus* are the most common fungi (Khurana et al., 2022). *Fusarium* is everywhere in our living environment. It is highly infectious and causes more serious consequences. It has a series of symptoms, including corneal tissue feathery margins, satellite lesions, immune ring, ulcers, perforations and even progression to endophthalmitis (Xie et al., 2006; Edelstein et al., 2012). *Fusarium* infection causes many immune responses in the body, such as macrophage activation, leukocyte infiltration and secretion of various inflammatory factors (Mitchell et al., 2007; Li et al., 2016). These biological processes will produce large amounts of reactive oxygen species (ROS), which will kill the *Fusarium*. Large amounts of ROS can cause damage to surrounding normal cells, promote inflammatory processes, and cause REDOX imbalance. Similar to other fungal keratitis, Fungal smear and culture is the gold standards (Sharma, 2012; Chidambaram et al., 2016). There are several approaches to treating *Fusarium* keratitis, such as topical and systemic antifungal drugs, laser therapy and surgery (Thomas and Kaliamurthy, 2013; Maharana et al., 2016; Prajna et al., 2016; Sharma et al., 2017). Although there are many treatments for *Fusarium* keratitis, the results are still poor and we still see many people who have lost their vision about it (Danielescu et al., 2022). Now, it is well established that multiple immune factors and enzymes were involved in the development of *Fusarium* keratitis, and they caused many different types of cell death. Ferroptosis, as an important mode of death, has not been reported in *Fusarium* keratitis (Mitchell et al., 2007; Karthikeyan et al., 2011; Hu et al., 2014; Li et al., 2016).

Ferroptosis is a new form of cell death. It is a non-apoptotic, iron-dependent form of regulated cell death (RCD) occurring. Its main feature is that lipid oxides can’t be metabolized by glutathione reductase reaction, and then Fe^2+^ oxidizes lipids to generate reactive oxygen species, thereby causing cellular damage. It is mainly due to the disruption of redox balance, the production of reactive oxygen species greater than antioxidants, which increases oxidation in the body and disrupts iron balance, causing an imbalance in cellular homeostasis and leading to cell death (Jiang et al., 2021). Ferroptosis is widespread in cellular metabolisms, such as substance metabolism (lipid metabolism, amino acid metabolism), cellular respiration, etc (Gao and Jiang, 2018; Lang et al., 2019). Ferroptosis is involved in the development of many diseases in current studies. These include infections, cancer, immune diseases, cardiovascular diseases, etc (Stockwell et al., 2017). Ferroptosis has become a new hot spot, and it provides a new pathway for the diagnosis and treatment of diseases (Weiland et al., 2019). Given the important role of oxidative stress in ferroptosis, we hypothesized that ferroptosis was involved in the development of *Fusarium* keratitis.

Currently, ferroptosis in *Fusarium* keratitis has not been studied. Therefore, we used data mining and data analysis approach to identify differentially expressed genes (DEGs) in corneal tissue between *Fusarium* keratitis and healthy people. Then, the DEGs intersected with the ferroptosis associated genes. We acquired ferroptosis related to DEGs (Ferr-DEGs). Meanwhile, we obtained hub genes and verified their association with immune infiltration. Finally, we verified the existence of this process by qPCR. Our study explained the potential role of ferroptosis in *Fusarium* keratitis and provides a new direction for treatment.

## Materials and methods

### Data source

**Figure 1:**
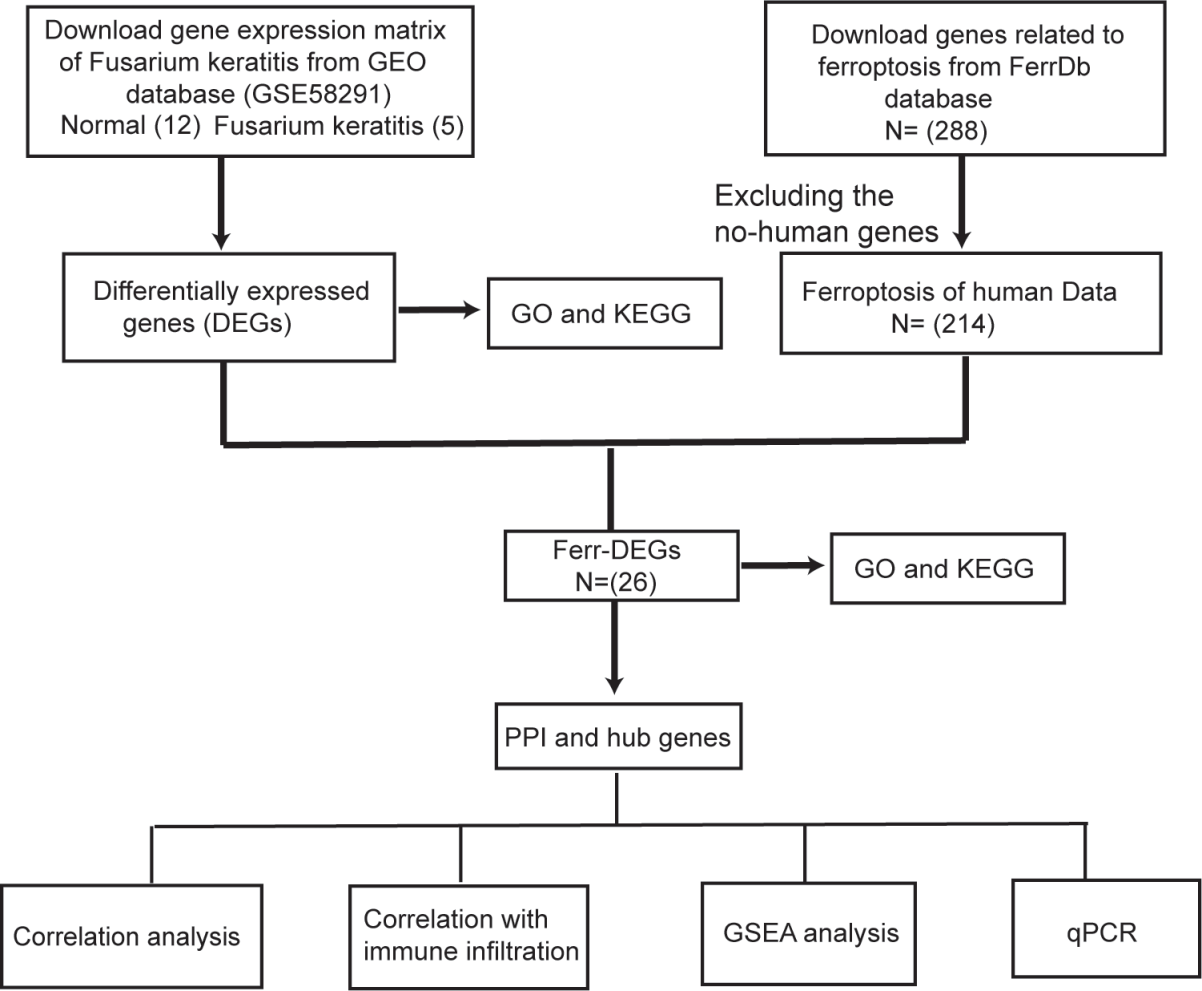
The analyses workflow flowchart.

The gene expression profile data (GSE58291) were obtained from the Gene Expression Omnibus (GEO) database (http://www.ncbi.nlm.nih.gov/geo). GSE58291 included 12 normal non-infected corneal tissues, 7 bacterial keratitis tissues, 5 ***Fusarium*** keratitis tissues, 2 ***Aspergillus*** keratitis tissues and 1 ***Lasiodiplodia*** keratitis tissue. 12 normal non-infected corneal tissues and 5 ***Fusarium*** keratitis tissues were selected for analysis. Ferroptosis-related genes were obtained from FerrDb (http://www.zhounan.org/ferrdb/legacy/index.html). It contained 288 genes associated with ferroptosis, including 108 drivers, 69 suppressors and 111 markers. However, we only selected 214 genes associated with Homo sapiens. The spearman’s method was used for data repeatability testing and differentially expressed genes (DEGs) correlation analysis, and the ggplot package on the R software was used for graphical plotting. Principal component analysis (PCA) was a method of regrouping the original variables into a new set of variables by dimensionality reduction, which can reflect as many characteristics of the original variables as possible using fewer dimensions. The DEGs were obtained through the limma package of the R software and the cutoff criteria for statistical significance were |logFC| > 1.0 and *p* value < 0.05. Then, we used the ggplot package to draw a heat map and a volcano map expressing the data. The flowchart for data processing is shown in **Figure 1**.

### Functional enrichment analysis

To obtain access to gene regulation and function, Gene Ontology (GO) and Kyoto Encyclopedia (KEGG) analyses were used. Functional enrichment analysis includes biological process (BP), cellular component (CC), molecular function (MF) and pathway analyses. The function was performed by the clusterProfiler package. We defined that the cutoff criteria for statistical significance were *p* value < 0.05. Then the results of the functional enrichment analysis were plotted by GOplot and ggplot packages in the R software.

### Protein-protein interaction network analysis and obtaining hub genes

PPI was composed by protein interaction to participate in various aspects of life processes. The string database was used and the minimum required interaction score was medium confidence (0.400) (https://cn.string-db.org/). Then PPI network was constructed by Cytoscape software (version 3.9.0). Next, important modules in PPI network were built by MCODE. It is a plugin in Cytoscape. The criteria for the MCODE analysis parameter setting: degree cut off = 2, MCODE scores > 5, max depth = 100, k-score = 2, and node score cutoff = 0.2. Finally, cytoHubba analysis was used for the search of 5 hub genes, it is also a plugin in Cytoscape. The top 5 hub genes in network ranked by MCC method.

### Enrichment analysis by gene set enrichment analysis

The GSEA version 4.1.0 software was performed for hub genes enrichment analysis. GSEA differs from the traditional enrichment function method in that it can determine the expression of a gene in different subgroups by samples of different subgroups and can obtain relevant enrichment information. First, the expression dataset file, phenotype labels file and gene sets file were loaded. Then, we needed to set the parameters. The database of C2.cp.kegg.v7.5.symbols.gmt (curated) was chosen. The number of permutations was 1000 and the permutation type was phenotype. Other settings are default. Gene pathways under pathways with |NES| > 1, *p* value < 0.05 and FDR < 0.25 were meaningful.

### Immune cell infiltration analysis

The CIBERSORT algorithm was used to calculate the immunological abundance of a sample in R software. It included the expression matrix of 22 immune cells in the annotation file LM22. Then, the immune cell abundance was calculated by the CIBERSORT R function. Another method for analyzing immune infiltration was the ssGSEA algorithm. It used the GSVA R package to calculate the abundance of 28 immune cell species in a single sample. We selected statistically significant results for analysis. We defined that the cutoff criteria for statistical significance were *p* value < 0.05.

### Cell culture and treatment

Human corneal epithelial cells (HCECs) were obtained from the CTCC (Jiangsu, China). HCECs were cultured in DMEM/F12 medium (Gibco) supplemented with 10% fetal bovine serum (Gibco), 1% Penicillin-Streptomycin (Gibco), Human Epidermal Growth Factor (hEGF) 10 ng/ml (Sigma Aldrich), 5ng/ml Insulin (Sigma Aldrich).

Deoxynivalenol (Sigma Aldrich) is one of the main toxins produced by *Fusarium*, which we used to simulate the effect of *Fusarium*. Group designs are shown as follows. The control group was treated with PBS; the DON group was treated with deoxynivalenol (10uM). Follow-up experiments were performed when the drug was treated for 24 hours.

### Quantitative polymerase chain reaction

Total RNA was extracted from the HCECs using Trizol and quantified by a spectrophotometer (ND 2000c, Thermo Scientific). The expression of hub genes was examined by real-time quantitative PCR (qPCR) using TB Green Premix (RR820A, Takara) and a real-time PCR system (CFX96, BioRad, United States). Experiment was repeated 3 times at least, with 3 sub-wells for each experiment. The primer sequences could be found in **Table 1**.

**Table 1:** Sequence of primers for qPCR.

Gene	Species	Direction	Primer sequence 5’→3’
HMOX1	Human	Forward	GCTGCTGACCCATGACA
		Reverse	AAGGACCCATCGGAGAA
GPX2	Human	Forward	GCTCTGAGGCACAACCAC
		Reverse	CCCAGGACGGACATACTT
ALOX5	Human	Forward	CACCAGACCATCACCCAC
		Reverse	AAGCACAGGGAGGCATAG
SRC	Human	Forward	GAGCGGCTCCAGATTGTCAA
		Reverse	CTGGGGATGTAGCCTGTCTGT
CYBB	Human	Forward	CAAGTGCCCAAAGGTGT
		Reverse	GAGAATGGATGCGAAGG
GAPDH	Human	Forward	GCTGAGTATGTCGTGGAG
		Reverse	CTTCTGAGTGGCAGTGAT

### Statistic

The data were analyzed using SPSS 22.0 (IBM, United States) and GraphPad (version 8.0.2). The normally distributed continuous variables were reported as means ± standard deviations, and Student’s *t*-test was used to see whether there was any significance between the means. Statistical significance was defined as a *p* value < 0.05.

## Results

### Validation of the datasets and DEGs identified *Fusarium* keratitis and normal cornea

**Figure 2:**
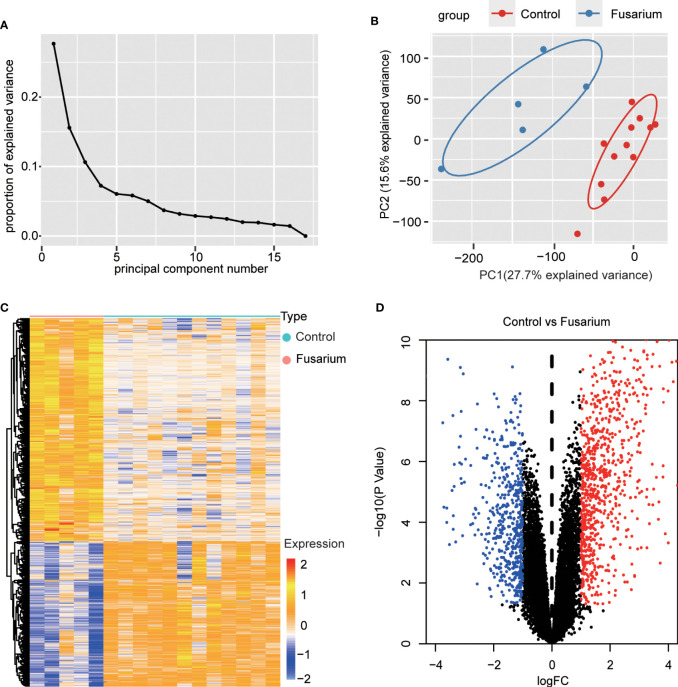
Data filtering and identification of DEGs in the corneal tissue samples. **(A)** The scree plots. **(B)** PCA of corneal tissue samples, *Fusarium* keratitis and normal non-infected corneal tissues. **(C)** Heat map for DEGs in *Fusarium* keratitis and normal non-infected corneal tissues. **(D)** The volcano plot. Red dots represent up-regulated genes, black dots represent not significant genes, and blue dots represent down-regulated genes. PCA, principal component analysis. DEGs, differentially expressed genes.

The expression profiling (GSE58291) was analyzed from the GEO database for this study. 12 normal non-infected corneal tissues and 5 *Fusarium* keratitis tissues were selected for analysis. First, the datasets were verified. The results of PCA showed good reproducibility of the data to be analyzed, and the differences within the group were small(**Figures 2A, B**). Then, the limma package in the R software was performed for differentially expressed genes. After screening with the threshold of |FC| > 1.0 and *p* < 0.05, 828 upregulated and 540 downregulated genes were determined for *Fusarium* keratitis tissues (**Table 2**). The volcano plot (**Figure 2C**) and heat map (**Figure 2D**) were displayed all the DEGs.

**Table 2:** Top thirty DEGs in *Fusarium* keratitis corneal tissue (GSE58291).

Gene Symbol	logFC	*P* Value	Level
MMP9	5.954909	6.92E-12	up
PI3	5.330881	6.78E-07	up
DEFB4	4.495306	2.43E-05	up
LOC728454	4.463143	3.74E-05	up
AQP9	4.460507	1.15E-06	up
LOC728835	4.31489	6.10E-06	up
FCN1	4.27841	5.00E-10	up
MARCO	4.170337	2.36E-09	up
RAC2	4.124469	5.39E-10	up
VCAN	4.043138	2.18E-12	up
ITGAM	4.027901	1.02E-10	up
IL1B	3.998764	0.000477	up
PLEK	3.992045	4.77E-09	up
CCL3L3	3.894214	0.000239	up
FCER1G	3.829228	4.04E-09	up
MMP9	5.954909	6.92E-12	down
INMT	-3.1886	3.38E-07	down
ANGPTL7	-3.30421	0.000444	down
SNORD3C	-3.3519	1.24E-06	down
OSAP	-3.35687	6.62E-06	down
GRP	-3.38533	6.11E-05	down
KRT12	-3.3895	0.000171	down
ADH1A	-3.40026	3.08E-08	down
C4orf49	-3.42878	8.99E-06	down
HES5	-3.5182	0.000542	down
ASIP	-3.57665	4.33E-10	down
CRTAC1	-3.60955	3.42E-05	down
DAPL1	-3.61124	0.000101	down
SCGB2A1	-3.71045	3.18E-05	down
EFHD1	-3.74771	5.32E-08	down

### DEGs functional annotation by the GO and KEGG analyses

**Figure 3:**
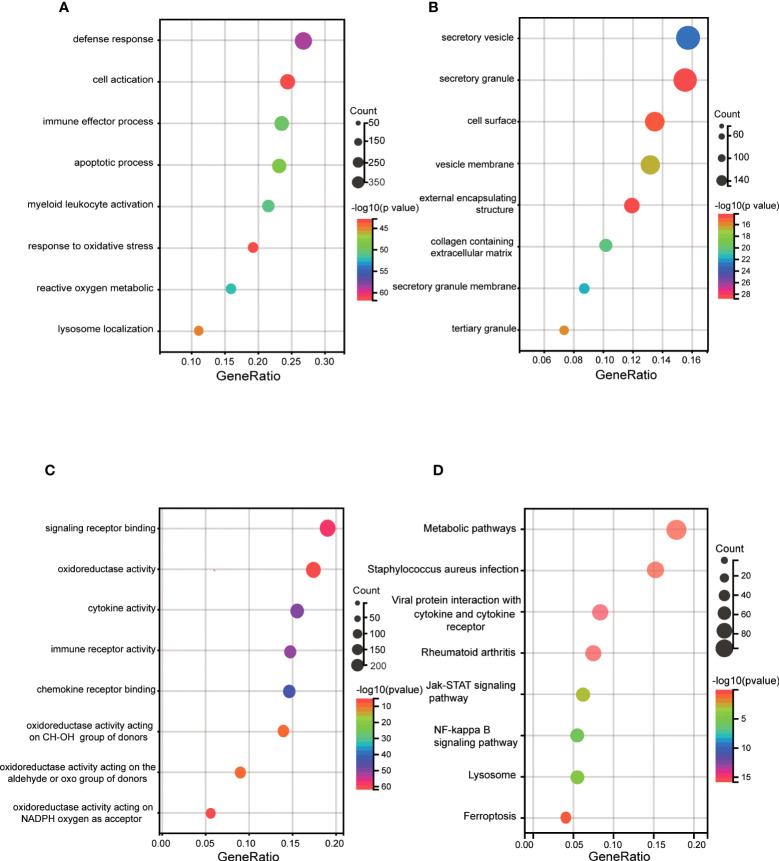
GO and KEGG enrichment analyses of DEGs. **(A)** Bubble plot of biological process of GO analysis. **(B)** Bubble plot of the cellular component of GO analysis. **(C)** Bubble plot of the molecular function of GO analysis. **(D)** Bubble plot of the KEGG pathway enrichment of DEGs. DEGs, differentially expressed genes; GO, Gene Ontology; KEGG, Kyoto Encyclopedia of Genes and Genomes.

First, enrichment analysis on the DEGs was performed by GO and KEGG. GO BP showed that they were mainly enriched in immune response, such as defense response, immune effector process and myeloid leukocyte activation. And apoptotic process, response to lipid, regulation of lipid metabolic process were also enriched (**Figure 3A**). CC involved specifically secretory vesicle, seceetory granule, cell surface, vesicle membrane, external encapsulating structure (**Figure 3B**). MF terms mainly contained signaling receptor binding, oxidoreductase activity, cytokine activity, immune receptor activity and terms related to oxidation reactions (**Figure 3C**). KEGG pathway mainly contained staphylococcus aureus infection, rheumatoid arthritis, viral protein interaction with cytokine rector, NF-κB signaling pathway, lysosome, JAK START-signaling pathway, ferroptosis and metabolic pathways (**Figure 3D**).

### Identification of data sets associated with ferroptosis

**Figure 4:**
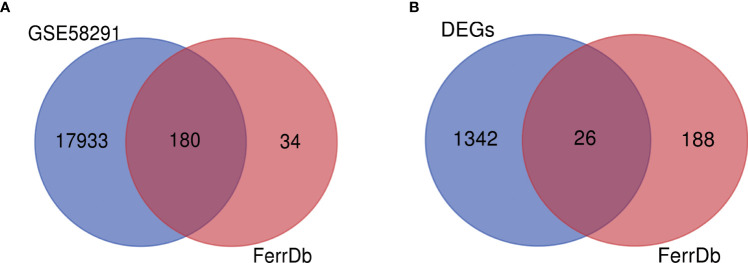
Identification of Ferr-DEGs in the *Fusarium* keratitis tissue. **(A)** The Venn diagram of gene expression profile data and ferroptosis-related genes. **(B)** The Venn diagram of DEGs and ferroptosis-related genes. Ferr-DEGs, ferroptosis related with differentially expressed genes; Ferr, ferroptosis; DEGs, differentially expressed genes.

To determine whether *Fusarium* keratitis is associated with ferroptosis, we intersected all expressed genes with ferroptosis database genes (All-Ferr). The Venn diagram showed that 180 genes crossed between DEGs. Only 34 ferroptosis-related genes were not among them (**Figure 4A**). Then, we took the intersection of the DEGs with the ferroptosis database and found that 26 genes intersected (**Ferr-DEGs**) (**Table 3**). The Venn diagram showed this result (**Figure 4B**).

**Table 3:** Ferroptosis differentially expressed genes of *Fusarium* keratitis.

Symbol	LogFC	*P* Value	Level
ALOX5	3.41743	3.11E-11	up
CYBB	3.091652	1.31E-11	up
NCF2	3.008236	2.53E-07	up
SLC2A6	1.843576	6.44E-06	up
PLIN2	1.77601	0.000561	up
HMOX1	1.681025	0.000551	up
MT1G	1.585544	0.012883	up
MT3	1.581896	0.000238	up
MAP3K5	1.553171	1.55E-08	up
SLC2A3	1.423861	0.001033	up
SOCS1	1.311836	2.20E-06	up
SRC	1.217767	1.32E-06	up
CA9	1.128851	5.15E-06	up
SLC2A14	1.062037	0.004004	up
ZFP36	-1.03998	0.024586	down
TF	-1.04383	0.006171	down
TSC22D3	-1.09449	1.18E-06	down
EGFR	-1.33943	2.86E-07	down
NNMT	-1.3507	0.001037	down
ATF3	-1.39368	0.042805	down
DUSP1	-1.5308	2.48E-05	down
NQO1	-1.87974	2.78E-07	down
AKR1C2	-1.97411	2.01E-07	down
PSAT1	-2.51174	5.12E-05	down
GPX2	-2.53092	0.000177	down
ANGPTL7	-3.30421	0.000444	down

### Validation and enrichment analysis of Ferr-DEGs

**Figure 5:**
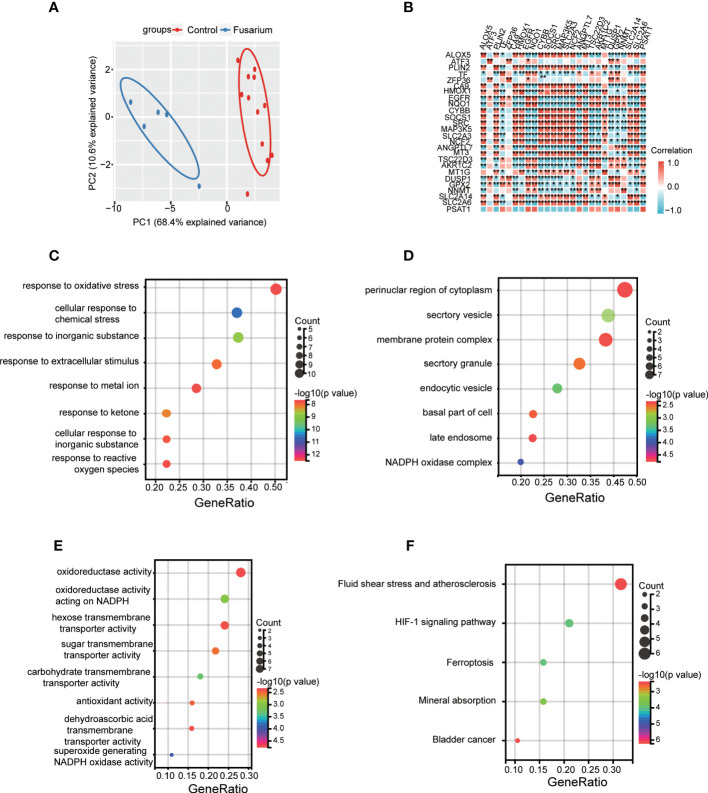
Data filtering and enrichment analyses of Ferr-DEGs. **(A)** PCA of Ferr-DEGs in 5 *Fusarium* keratitis samples and 12 normal samples. **(B)** Correlation heat map of Ferr-DEGs. **(C)** Bubble plot of biological process of GO analysis. **(D)** Bubble plot of the cellular component of GO analysis. **(E)** Bubble plot of the molecular function of GO analysis. **(F)** Bubble plot of the KEGG pathway enrichment of Ferr-DEGs. Ferr-DEGs, ferroptosis related to differentially expressed genes; Ferr, ferroptosis; DEGs, differentially expressed genes; GO, Gene Ontology; KEGG, Kyoto Encyclopedia of Genes and Genomes; CA, principal component analysis. *p < 0.05, **p < 0.01.

Ferr-DEGs were validated. The results of PCA showed that Ferr-DEGs had good reproducibility and small outliers of these genes within the group (**Figure 5A**). Spearman correlation analysis proved that these genes were well correlated (**Figure 5B**). Next, enrichment analysis on the Ferr-DEGs was performed (**Table 4**). GO BP was mainly enriched in the reaction with oxidative stress and response to metal ion (**Figure 5C**). CC was mainly enriched in NADPH oxidase complex and endocytic vesicle (**Figure 5D**). MF was mainly enriched in oxidoreductase activity and nutrient metabolism (**Figure 5E**). The results of KEGG were also mainly reactive oxygen species, HIF-1 signaling pathway, ferroptosis and so on (**Figure 5F**).

**Table 4:** Analysis of GO and KEGG enrichment of Ferr-DEGs.

Term	Description	*P* Value	GeneID
GO : BP	response to oxidative stress	3.20E-13	ALOX5/HMOX1/EGFR/NQO1/CYBB/SRC/MAP3K5/NCF2/ANGPTL7/MT3/DUSP1/GPX2
GO : BP	cellular response to chemical stress	2.59E-11	ALOX5/HMOX1/EGFR/NQO1/CYBB/SRC/MAP3K5/NCF2/MT3/GPX2
GO : BP	response to inorganic substance	1.96E-09	TF/HMOX1/EGFR/NQO1/CYBB/SRC/MAP3K5/MT3/MT1G/DUSP1
GO : BP	cellular response to cadmium ion	2.06E-09	HMOX1/EGFR/CYBB/MT3/MT1G
GO : BP	response to ketone	8.25E-09	CA9/EGFR/NQO1/CYBB/SRC/AKR1C2/DUSP1
GO : CC	perinuclear region of cytoplasm	1.67E-05	ALOX5/TF/HMOX1/EGFR/CYBB/SRC/MT3
GO : CC	nadph oxidase complex	0.000100258	CYBB/NCF2
GO : CC	endocytic vesicle	0.000524138	TF/EGFR/CYBB/NCF2
GO : CC	secretory vesicle	0.001115424	ALOX5/TF/CYBB/SLC2A3/NCF2/MT3
GO : CC	secretory granule	0.003391582	ALOX5/TF/CYBB/SLC2A3/NCF2
GO : MF	hexose transmembrane transporter activity	4.05E-06	SLC2A3/SLC2A14/SLC2A6
GO : MF	sugar transmembrane transporter activity	6.98E-06	SLC2A3/SLC2A14/SLC2A6
GO : MF	oxidoreductase activity acting on nadph	1.59E-05	NQO1/CYBB/NCF2/AKR1C2
GO : MF	carbohydrate transmembrane transporter activity	2.33E-05	SLC2A3/SLC2A14/SLC2A6
GO : MF	dehydroascorbic acid transmembrane transporter activity	4.64E-05	SLC2A14/SLC2A6
KEGG	Fluid shear stress and atherosclerosis	0.006171	HMOX1/NQO1/SRC/MAP3K5/NCF2/DUSP1
KEGG	Ferroptosis	1.18E-06	TF/HMOX1/CYBB
KEGG	HIF-1 signaling pathway	2.86E-07	TF/HMOX1/EGFR/CYBB
KEGG	Mineral absorption	0.001037	TF/HMOX1/MT1G
KEGG	Bladder cancer	0.042805	EGFR/SRC

### Identification of the hub genes of Ferr-DEGs

**Figure 6:**
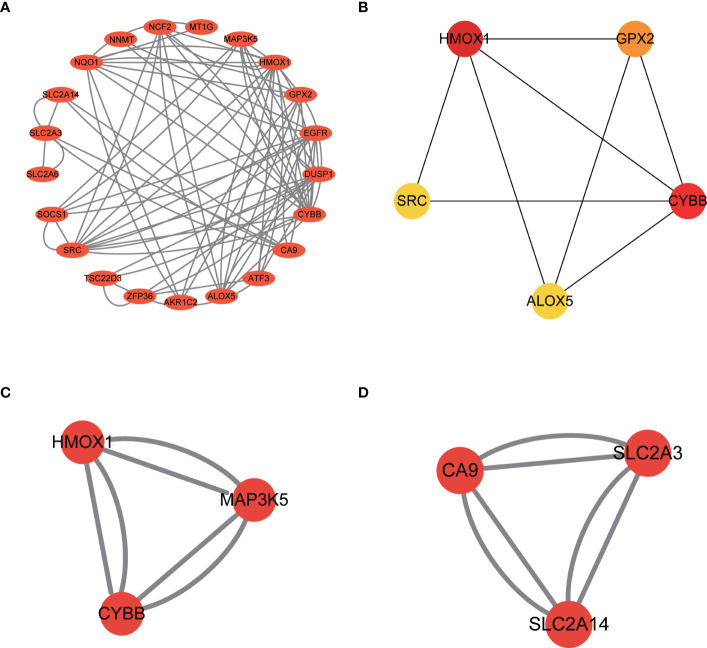
Identification of Ferr-DEGs hub genes in the *Fusarium* keratitis tissue. **(A)** Construction of a PPI network of Ferr-DEGs. **(B)** The top 5 hub genes of Ferr-DEGs. **(C)** MCODE 1 of the hub genes. **(D)** MCODE 2 of the hub genes. Ferr-DEGs, ferroptosis related to differentially expressed genes; Ferr, ferroptosis; DEGs, differentially expressed genes; PPI, protein-protein interaction.

Import the Ferr-DEGs into the string website to get the PPI network (**Figure 6A**). The PPI network was constructed with Cytoscape. The top 5 hub genes were identified with the cytoHubba plug-in. The hub genes included HMOX1, CYBB, GPX2, ALOX5 and SRC (**Figure 6B**). Next, the MCODE plug-in was used to analyze the PPI network and the two groups’ scores > 3. MCODE 1 included HMOX1, MAP3K5, CYBB (**Figure 6C**). MCODE 2 included CA9, SLC2A3, SLC2A14 (**Figure 6D**). Ultimately, both HMOX1 and CYBB played an important role in the CytoHubba and MCODE analyses.

### GSEA analysis of the top 5 hub genes

**Figure 7:**
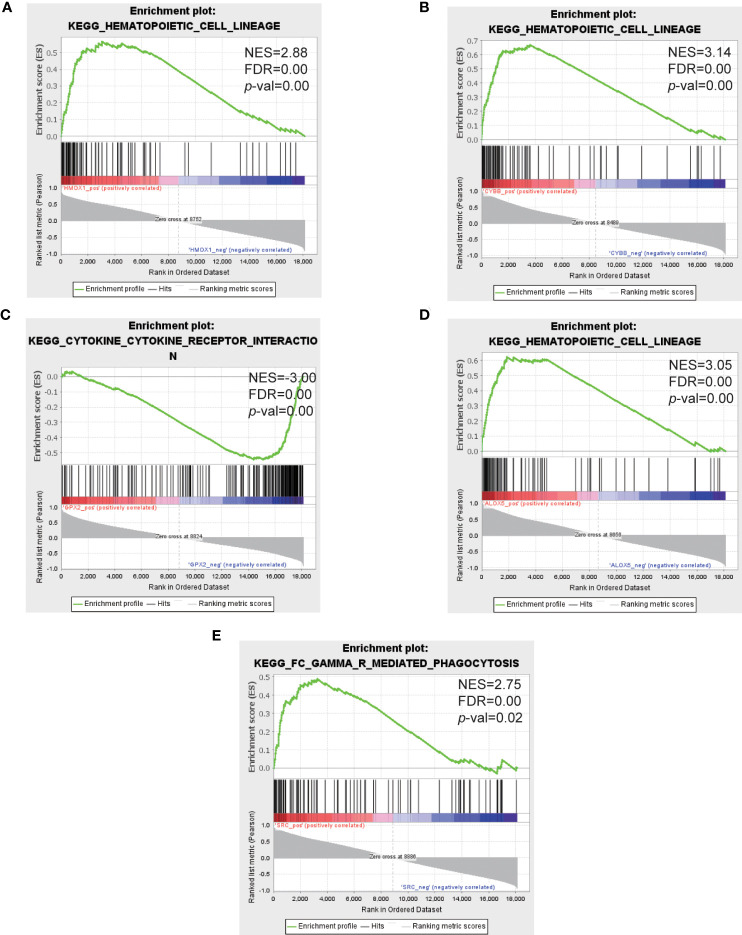
GSEA analysis of the top 5 hub genes. **(A–E)** Represent the pathways about function enrichment of HMOX1, CYBB, GPX2, ALOX5 and SRC. GSEA, gene set enrichment analysis.

The top 5 hub genes were entered into GSEA software for enrichment analysis at *p* value < 0.05. Based on the expression of each hub gene in the expression profile, we analyzed the pathways in which they are located using the KEGG pathway database. The results showed that the enriched pathways mainly concentrated in hematopoietic cell lineage, lysosome, FC gamma R mediated phagocytosis (**Figures 7A–E**).

### Identification of differences in immune infiltration between corneal tissues

**Figure 8:**
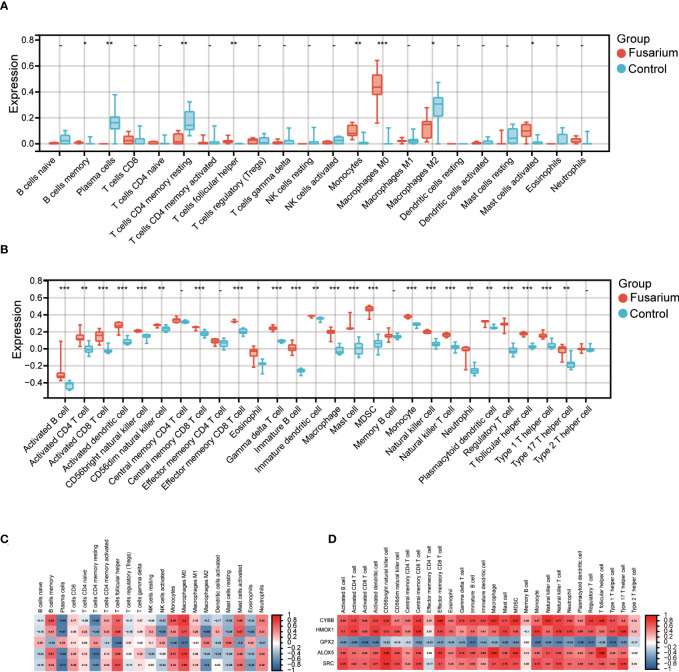
Immune infiltration of the samples and correlation with hub genes. **(A)** Box plot showing the immune cell heterogeneity of the two sample groups by CIBERSORT algorithm. **(B)** Box plot showing the immune cell heterogeneity of the two sample groups by ssGSEA algorithm. **(C)** Representative correlation heatmap between the result of CIBERSORT algorithm and the hub genes. **(D)** Representative correlation heatmap between the result of ssGSEA algorithm and hub genes. Data are shown as mean ± SD. *p < 0.05, **p < 0.01, ***p < 0.001.

To identify the differences in immune infiltration between the two tissues, we used CIBERSORT and ssGSEA algorithm for analysis. The B cell memory, T cells follicular helper, monocytes, macrophages M0 and mast cells activated in *Fusarium* keratitis tissues were higher than normal corneal tissues in the CIBERSORT algorithm (**Figure 8A**). In the spearman correlation analysis, the hub genes were highly correlated with the above immune cells. We could see that all of them were positively correlated except for GPX2 (**Figure 8C**). In the results of ssGSEA, immune cell infiltration in *Fusarium* keratitis tissues were significantly higher than in normal corneal tissues except for central memory CD4 T cell, effector memory CD4 T cell, memory B cell and type 2 T helper cell (**Figure 8B**). The correlation between hub genes and ssGSEA results was similar to that of CIBERSORT, except that GPX2 was negatively correlated, while all others were positively correlated (**Figure 8D**). In summary, we could see that T cells follicular helper, monocytes macrophages and mast cells activated played an important role in *Fusarium* infection, and they had a high correlation with hub genes.

### Validation of hub genes associated with ferroptosis in *Fusarium* keratitis

**Figure 9:**
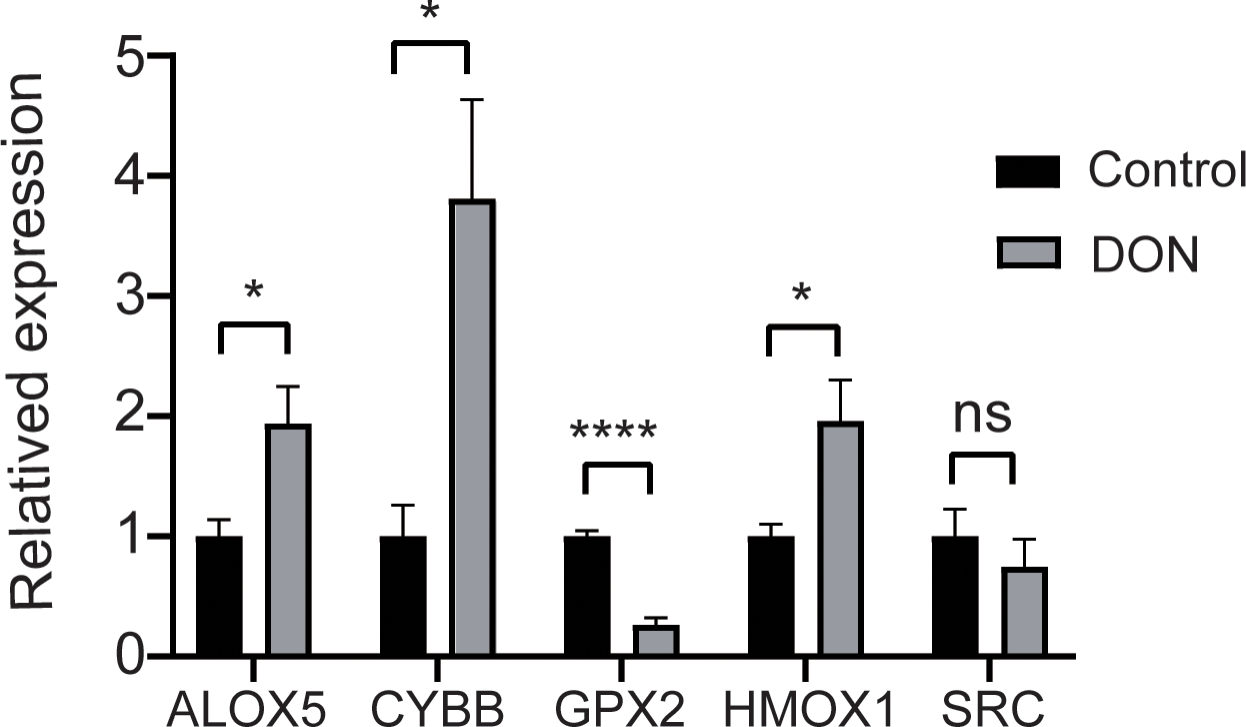
Validation the expression of hub genes. qPCR results for mRNA levels of the top 5 hub genes HMOX1, CYBB, GPX2, ALOX5 and SRC. Data are shown as mean ± SD. *p < 0.05, ****p < 0.0001, ns, no significance.

The qPCR was used to verify the mRNA of the top 5 hub genes. HMOX1, CYBB, ALOX5 were higher expressed in the DON group than in the control group. GPX2 was lower expressed than the control group compared with the DON group and only the expression of SRC showed no significant differences with the DON group (**Figure 9**).

## Discussion

Our analyses suggested that ferroptosis was activated during *Fusarium* keratitis. At the same time, we obtained the relevant hub genes and analyzed them. It provided a new direction for the treatment of *Fusarium* keratitis. We got 1368 DEGs, including 828 upregulated and 540 downregulated genes from the datasets GSE58291. By KEGG enrichment analysis, we found that ferroptosis plays an important role in *Fusarium* keratitis. Then we got 26 intersecting genes from the intersection of DEGs and FerrDb. By GO and KEGG enrichment analysis, they were mainly associated with oxidative stress and ferroptosis, then hub genes were obtained. The top 5 genes with the highest scores were selected, which are HMOX1, CYBB, GPX2, ALOX5 and SRC. Analysis of hub genes using GSEA software further suggested the role of ferroptosis in *Fusarium* keratitis. Then the samples were analyzed for immune heterogeneity and their correlation with hub genes. Finally, the expression of the mRNA of hub genes were demonstrated by the experiment. SRC expression showed no statistically significant difference. So SRC might not serve as the hub gene.

Ferroptosis is a new type of cell death that is different from other cell death processes. It is mainly depending on Fe^2+^. It catalyzes the high expression of unsaturated fatty acids on cell membranes through Fenton reaction and peroxidation reaction, and lipid peroxidation occurs and induces cell death. At the same time, the lack of reducing substances is also an important factor. Lipid peroxidation and ROS production are important features of ferroptosis. It is regulated by a variety of cellular activities including redox homeostasis, iron handling, mitochondrial activity and metabolism of amino acids, lipids and sugars, in addition to various signaling pathways relevant to disease (Gao and Jiang, 2018; Jiang et al., 2021). Ferroptosis is associated with many diseases, including cancer, blood diseases, brain and neurodegenerative diseases, heart diseases and infectious diseases (Qiu et al., 2020). It is well known that *Fusarium* keratitis activated a variety of immune cells and enhances oxidative stress in corneal tissue. It could stimulate neutrophils to produce pro-inflammatory factors, which then promote the production of reactive oxygen species by epithelial cells (Ruban et al., 2018). Some studies have also shown that the *Candida albicans* or *Aspergillus fumigatus* can infect alveolar macrophages and may trigger feroptosis in cells. (Bagayoko and Meunier, 2022). In our findings, we enriched many ferroptosis-associated functions and pathways including response to oxidative stress, response to metal ion, oxido reductase activity acting on NADPH, regulation of lipid metabolic process, and so on. *Fusarium* keratitis is an infectious disease that causes cell death by activating multiple immune responses, producing ROS, increasing ROS, and disrupting the metabolism of multiple substances. As a result, we could hypothesize that disturbance of iron and ROS metabolism were a major important cause of corneal tissue damage in *Fusarium* keratitis patients.

The HIF-1 signaling pathway associated with ferroptosis in *Fusarium* keratitis was discovered using GO, KEGG, and GSEA enrichment analysis. HIF-1 (hypoxia-inducible factor 1), the transcriptional activator that functions as a master regulator of oxygen homeostasis. We proposed the following model based on our data. When *Fusarium* infected the cornea, it caused local hypoxia and mitochondrial generation of superoxide in the corneal epithelial tissue. Then, HIF-1 was activated (Semenza, 2001). Next, HIF-1 would induce the expression of HO-1. Then HO-1 increased heme degradation, releasing iron ions from the heme, and the increased iron promotes ferroptosis (Lei et al., 2019). So, in the GSEA enrichment analysis, the pathway to hematopoietic cells was enriched. On the other hand, we found that the NF-κB signaling pathway might also be associated with ferroptosis. Nuclear factor-κB (NF-κB) consists of multiple transcription factors. It is associated with a variety of biological processes, including inflammation, immunity, cell proliferation, differentiation, and survival (Oeckinghaus and Ghosh, 2009). *Fusarium* infection of corneal tissues might induce the release of inflammatory factors and activate the NF-κB signaling pathway. The current study finds that inhibition of RSL3 activates the NF-κB signaling pathway and can induce ferroptosis (Li et al., 2021). Beyond the initial, in the results of GSEA enrichment analysis, lysosomes were shown to be involved in *Fusarium* keratitis ferroptosis. Some studies have proven that lysosome inhibitors could inhibit ROS generation and local iron accumulation can be involved in ROS generation within lysosomes (Dixon et al., 2012). Erastin induce enhanced lysosomal membrane permeabilization and lysosomal disruption, leading to ferroptosis (Zhou et al., 2020).

In the present study, we obtained the top5 hub genes. We verified the differential expression of hub genes in HCECs by qPCR. HMOX1 (heme oxygenase 1) is an enzyme in the catabolism of heme and catalyzes the degradation of heme to biliverdin (Hassannia et al., 2019). In fungal keratitis, HMOX1 can be increased and suppressed levels of antioxidant enzymes, inducing ferroptosis (Hua et al., 2017). CYBB (Cytochrome B-245 Beta Chain) can transport electrons through the plasma membrane to generate ROS and activate ferroptosis (Yang et al., 2020). However, the exact mechanism by which it activates ferroptosis in *Fusarium* keratitis was currently unclear and needed to continue to explore. GPX2 (glutathione peroxidases2) is a member of the peroxidase family and similar to GPX4 in that it is an antioxidant and anti-inflammatory enzyme. In addition to serving as an antioxidant function, it also has anti-apoptotic, anti-inflammatory, and functions that are closely related to tumor cell growth, invasion, and metastasis (Brigelius-Flohé and Flohé, 2020). Current studies have shown that fungal keratitis can cause an experimental inflammatory response in the corneal tissue, resulting in a decrease in the levels of cyclooxygenase-2 (COX2), superoxide dismutase (SOD-1), and glutathione peroxidase (GPX2) in human corneal epithelial cells (HCECs), as well as activation of ferroptosis (Qin et al., 2022). ALOX5 (arachidonate 5-lipoxygenase) is a protein that is required for the activation of the lipid peroxidation reaction. It is an iron-containing enzyme that is associated with several modes of cell death. It currently activates ferroptosis primarily by promoting inflammatory responses as well as lipid peroxidation pathways (Shi et al., 2013; Sun et al., 2019).

In *Fusarium* keratitis, the immune response is an important biological process. In the enrichment analysis, we enriched many immune-related pathways, and we also analyzed the abundance of immune cells. T cells follicular helper, monocytes, macrophages and mast cells played an important role. This was essentially the same as the previous study. The main role of macrophages is to engulf useless cells and dead cells. And in fungal keratitis, it can inhibit spore germination and kill spores or mycelium of fungi (Zhang et al., 2009). Xie et al. (Xie et al., 2018). demonstrate the role of mast cells in fungal keratitis by mast cell inhibitors. The role of T cells follicular helper and monocytes in fungal keratitis was poorly documented. There have been numerous studies showing a correlation between ferroptosis and immune response in infectious diseases. For example, the macrophages are less effective in phagocytosing ferroptotic Jurkat T cells as compare with their apoptotic counterparts (Klöditz and Fadeel, 2019); T cells and helper T cells are regulated by lipid peroxidation and ferroptosis (Funes et al., 2018). Therefore, we further examined the correlation between hub genes and infiltrating immune cells. Macrophages can regulate the dynamic balance of iron by removing aging red blood cells. This might induce ferroptosis in macrophages, thereby limiting their immune activity. For example, long-term storage of red blood cells in the refrigerator leads to increased phagocytosis, which is inhibited by the iron sag inhibitor ferrostatin-1 in splenic red marrow macrophages (Youssef et al., 2018). To my surprise, the neutrophil difference was not statistically significant in the CIBERSORT algorithm. Many studies have demonstrated that neutrophils are the main immune cells in early *Fusarious* keratitis (Karthikeyan et al., 2011; Leal et al., 2012). Bridget Ratitong et al. (Ratitong and Pearlman, 2021). have summarized these studies. The neutrophil system generates O^2−^, hydrogen peroxide (H_2_O_2_) and hydroxyl radical. They can generate ROS (Urban and Backman, 2020). The ROS might activate ferroptosis. The main reason for calculation of neutrophils might be a problem with the CIBERSORT algorithm calculation formula. CIBERSORT is a tool for deconvolution of expression matrices of immune cell subtypes based on the principle of linear support vector regression. It depends primarily on fidelity of reference profiles (Newman et al., 2015). Perhaps because there were too few reference documents, resulting in inaccurate results. However, the molecular mechanism between ferroptosis and immunity remains unclear.

Although we used many bioinformatics analysis methods and statistical methods to analyze fungal keratitis. However, we should also be aware of the shortcomings. Firstly, the sample size of this study was not sufficiently large. Second, this study was retrospective, so clinical data were lacking. Thirdly, the data in this study relied on the GEO database, and we would integrate data from other sources in future studies. Finally, the study used DON to simulate *Fusarium* infection, which had some limitations, and in future studies, we would improve the experimental protocol to make the analysis more convincing.

## Conclusion

In this study, we suggested several hub genes and pathways that are closely associated with ferroptosis in *Fusarium* keratitis. These results contribute to a better understanding of ferroptosis in *Fusarium* keratitis and provide us with new directions for the treatment of *Fusarium* keratitis. Unfortunately, our samples are not large enough and the specific mechanism is not clear enough, further research is needed.

## Data availability statement

Publicly available datasets were analyzed in this study. This data can be found here: https://www.ncbi.nlm.nih.gov/gds/?term=gse58291 http://www.zhounan.org/ferrdb/.

## Author contributions

XT provided idea. XT, XX, XS and YT analyzed the data and performed the experiments. YL, JL, and YD wrote the manuscript. JZ and LL assisted in revising the manuscript. LL gave important guidance and analysis in the review process. All authors contributed to the article and approved the submitted version.
